# Targeting N-Terminal Huntingtin with a Dual-sgRNA Strategy by CRISPR/Cas9

**DOI:** 10.1155/2019/1039623

**Published:** 2019-11-16

**Authors:** Junjiao Wu, Yu Tang, Chun-Li Zhang

**Affiliations:** ^1^National Clinical Research Center for Geriatric Disorders, Department of Geriatrics, Xiangya Hospital, Central South University, Changsha, Hunan Province, China; ^2^Department of Rheumatology and Immunology, Xiangya Hospital, Central South University, Changsha, Hunan Province, China; ^3^Department of Molecular Biology, University of Texas Southwestern Medical Center, Dallas, TX, USA; ^4^Hamon Center for Regenerative Science and Medicine, University of Texas Southwestern Medical Center, Dallas, TX, USA

## Abstract

Huntington's disease (HD) is an autosomal dominant progressive neurodegenerative disorder, caused by a CAG/polyglutamine (polyQ) repeat expansion in the *Huntingtin* (HTT) gene. The polyQ tract is located in and transcribed from N-terminal HTT of exon 1. HTT is a large multifaceted protein, which participates in a range of cellular functions. Previous studies have shown that truncated HTT, which lacks N-terminus, retains specific functions that can produce neuroprotective benefits. It gives an insight that it is possible to repair HD by removing deleterious N-terminal HTT with CRISPR/Cas9, without compromising functions of remaining HTT peptides. To successfully generate functional truncated HTT proteins, an alternative downstream ATG start codon that is capable of initiating truncated HTT expression is required. In this study, we searched all possible in-frame ATGs before exon 7 and demonstrated that one of them can indeed initiate the downstream GFP expression in plasmids. We then tried to remove endogenous N-terminal HTT with an optimized dual-sgRNA strategy by CRISPR/Cas9; however, we cannot detect obvious traits of truncated HTT expression. Our results suggest that noncanonical ATGs of N-terminal HTT may not be effective in the genomic context, as in the construct context. Nevertheless, our study examined the therapeutic efficacy of downstream noncanonical ATGs for protein translation and also provided an optimized dual-sgRNA strategy for further genome manipulation of the HTT gene.

## 1. Introduction

Huntington's disease (HD) is an autosomal dominant neurodegenerative disease caused by the expanded CAG tract resided in the first exon of the *Huntingtin* (HTT) gene [1, 2]. Basically, the pathogenic mutant HTT consists of more than 35 CAG repeats, which are then translated into polyglutamine (polyQ) proteins [3, 4]. Although HTT is ubiquitously expressed in the body, expanded polyQ proteins may form intracellular aggregates and preferentially cause the loss of medium spiny neurons (MSNs) in the striatum, via a gain of toxic function [5]. The CAG tract length determines HTT propensity for aggregation and toxicity and is inversely correlated with age of onset in HD patients [6].

HTT is a multiple conformation protein of normally 3144 amino acids and has conserved N-terminal sequences [7, 8]. By binding with other proteins, HTT is considered to be multifaceted that is essential for a spectrum of cellular functions, such as embryonic development, antiapoptotic pathway, BDNF modulation, ciliogenesis, autophagy, and vesicular transport [7]. N-terminal HTT containing the first 17 amino acids (N17) and the polyQ tract can be formed by proteolytic cleavage and is one of the most widely studied HTT peptides. HTT proteolysis in patient brains is the cascade of various cleavage events. Numerous proteases, including caspases, calpains, cathepsins, matrix metalloproteinases, and aspartic proteases, that cleave HTT have been reported [9–13].

The N17 domain, as an evolutionarily conserved domain among vertebrates, functions as a nuclear export signal (NES) [14] and is necessary for nuclear exclusion of small mutant HTT fragments, thereby modifying nuclear pathogenesis and disease severity [15]. The N17 domain forms an amphipathic *α*-helical structure that signals endoplasmic reticulum (ER) stress [16] and also senses reactive oxygen species (ROS) stress, specifically through direct oxidation of a highly conserved methionine at position 8 [17]. Moreover, the polyQ tract can act as a flexible domain, allowing the flanking domains to come into close spatial proximity to form proper functional conformations [18]; however, this flexibility may be impaired with expanded polyQ tracts. Importantly, multiple posttranslational modifications, such as phosphorylation, ubiquitination, acetylation, and sumoylation, have been identified in the N17 domain, that may affect the clearance of HTT and its subcellular localization [19–21]. Thus, N-terminal HTT is considered to be important in regulating protein aggregation, subcellular localization, and cytotoxicity. Despite of this fact, other modification sites in the remaining HTT protein have been demonstrated to possess pivotal cellular functions as well [7]. For instance, sequential N-terminal proteolysis of HTT also generates a C-terminal fragment, which induces cytotoxicity via abnormal vacuolation of ER and increases ER stress [22]. Interestingly, without a whole stretch of N-terminus, however, HTT may also retain some beneficial functions [23, 24]. For example, Liu et al. reported that a truncated HTT lacking the first 237 amino acids spanning exon 1∼6 can rescue the HTT deficiency-induced neuritic degeneration, which largely depends on the dynein-binding region known to be involved in intracellular trafficking [24].

So far, different strategies have been employed to produce therapeutic effects in both cellular and animal models of HD, including traditional avenues such as RNA interference (RNAi) [25–27] and antisense oligonucleotides (ASOs) [28–31], pharmacological treatments [32, 33], and the emerging genome editing by CRISPR/Cas9 both in vitro and in vivo [34–39]. A recent study by Southwell et al. developed ASOs targeting HD single-nucleotide polymorphisms (SNPs) that can selectively suppress mutant HTT expression in the mouse HD model [31]. They also achieved preclinical efficacy data of those HTT ASOs delivered to cynomolgus monkeys via lumbar puncture [31]. Similarly, two other recent studies have used an allele-selective CRISPR/Cas9 strategy based on protospacer adjacent motif (PAM) sites created by altering SNPs, thereby selectively inactivating mutant HTT allele for a given diplotype [34, 35]. As this is a quite novel personalized strategy, it requires a comprehensive analysis of patients' HTT haplotype patterns. The nonallele-selective inactivation of HTT has also been achieved by using a pair of sgRNAs flanking CAG repeats, in a transgenic HD mouse model [36], as well as in patient-derived fibroblasts with the help of Cas9 nickase [37]. CRISPR/Cas9 was also employed to correct the HTT mutation in an HD-induced pluripotent stem cell (iPSC) line, which can rescue the HD phenotypes such as impaired neural rosette formation, increased susceptibility to growth factor withdrawal, and deficits in mitochondrial respiration [38]. Notably, CRISPR/Cas9 can permanently disrupt the targeted genes, rendering it to be a more efficient therapeutic approach than those with continuous delivery of drugs for treatment. However, it also brings about the caveats that the treatment cannot be reversed in the event of adverse effects.

Taking advantage of the higher efficiency by the CRISPR/Cas9 system, we hypothesize that we may remove a length of N-terminal HTT to abolish the neurotoxicity stressed by polyQ aggregations and meanwhile keep the functions of probably generated truncated HTT after CRISPR targeting. To successfully obtain this type of endogenous truncated HTT, it is required that an alternative downstream ATG start codon will be capable of initiating truncated HTT translation, albeit at lower levels. Therefore, in this study, we tried to search all possible in-frame ATGs within the first 6 exons that coded for 249 amino acids, which were then cloned to fuse with GFP in plasmids, and we demonstrated that one of them can indeed start downstream translation. However, after removing endogenous N-terminal HTT by an optimized dual-sgRNA targeting, we failed to detect obvious traits of truncated HTT expression. Our results suggest that noncanonical ATGs of N-terminal HTT may not be effective in the genomic context, as in the construct context.

## 2. Materials and Methods

### 2.1. Cloning and Sequencing

Constructs of E3a-, E3b-, E4-, and E6-GFP were generated based on the pcDNA3.1(+) vector using Gibson Assembly Master Mix (New England Biolabs, MA, USA). Specifically, different lengths of HTT fragments were amplified from a human cDNA library with 21 CAGs located in N-terminus. E3a∆-, E3b∆-, E4∆-, and E6∆-GFP plasmids were then, respectively, constructed by PCR using primers flanking exon 2 and 5′UTR from their corresponding plasmids and finally self-ligated into circle plasmids. The length of 5′UTR in all constructs is 145 bp, which is exactly the full-length of 5′UTR in the NM_002111 transcript. All pcDNA constructs were finally confirmed by Sanger sequencing. For genome editing, lentiCRISPRv2 (Addgene #52961) was used as the backbone, and sgHTT sequences were designed with the help of CHOPCHOP website (http://chopchop.cbu.uib.no/). In brief, sgRNA oligos were annealed and inserted into the BsmBI digested vector. Junctions of exon 1/2 or targeted genomic regions were amplified by PCR and ligated into the pMD20T vector (Takara Bio Inc., CA, USA) for T-A cloning. White colonies were picked out for plasmid extraction and Sanger sequencing. Sequences of sgHTT and PCR primers are listed in Table 1.

### 2.2. Cell Culture

HEK293 cells were maintained in the DMEM medium (Thermo Fisher Scientific, MA, USA) supplemented with 10% fetal bovine serum (Corning, NY, USA) and 1% penicillin/streptomycin at 37°C and 5% CO_2_. For transfection, passage and seed HEK293 cells at a density of 2 × 10^5^ cells per well of a 6-well plate. The same amount of 0.5 *μ*g constructed pcDNA plasmids were then transfected by the polyethylenimine (PEI) reagent. GFP signals and protein aggregations were observed 24 hrs later. For CRISPR targeting, HEK293 cells were transfected with different sgRNA pairs (0.5 *μ*g/0.5 *μ*g mixture) by the PEI reagent. After 48 hrs, collect HEK293 cells for indel analysis by the T7E1 assay. For clonal cell line selection, passage targeted cells with 0.25% trypsin and further dilute and seed 6000 single cells in a 10 cm dish. Single colonies were picked out under a microscopy and further examined by junction PCR. PCR primers are listed in Table 1.

### 2.3. T7E1 Assay

The cleavage efficiency of dual sgHTT was examined by the T7E1 assay accordingly to the previously reported method [40]. In brief, transfected cells were digested and spun down at 250 g for 4 min and later lysed with DNA lysis buffer (100 mM Tris-HCl (pH 8.0), 25 mM EDTA (pH 8.0), 100 mM NaCl, 1% Triton X-100, and 50 ng/*μ*l RNase A) containing 0.5 mg/ml Proteinase K at 55°C for overnight. DNA lysates were used directly as templates for genomic PCR. T7E1 primers are listed in Table 1. PCR products were then melted and reannealed to form heteroduplexes, which were then digested by T7E1 (New England Biolabs) and electrophoresed in 2% agarose gels. Calculate the fraction of the PCR product cleaved (*f*_cut_) by using the following formula: *f*_cut_ = (*b* + *c*)/(*a* + *b* + *c*), where a is the integrated intensity of the undigested PCR product and *b* and *c* are the integrated intensities of each cleavage product. Indel occurrence was estimated with the following formula: indel (%) = 100 × (−$\sqrt{\left(1-f_{\text{cut}}\right)}$).

### 2.4. Gene Expression

Gene expression was performed accordingly to the previously reported method [41]. Total mRNAs were isolated from cultured cells using TRIzol (Invitrogen, Carlsbad, CA, USA) and were reverse transcribed into cDNAs using a SuperScript III kit (Invitrogen). Quantitative PCR was then performed using SYBR GreenER SuperMix (Invitrogen) on an ABI 7900HT thermocycler (Applied Biosystems). The PCR program consisted of 2 min at 50°C and 10 min at 95°C, followed by 40 cycles of 15 sec at 95°C and 45 sec at 62°C. Relative expression levels were analyzed using the 2^−ΔΔCt^ algorithm normalized to the housekeeping gene GAPDH. Primer sequences are listed in Table 1.

### 2.5. Western Blot

Total proteins were extracted from cultured cells and homogenized mice brain tissues, respectively, with RIPA lysis buffer. Proteins were then separated by SDS-PAGE with Tris-glycine gels (8% for HTT; 15% for GFP) and transferred onto PVDF membranes (Millipore, MA, USA). Nonspecific sites were blocked in 5% nonfat milk for 1 hr at room temperature. The blots were then probed at 4°C for overnight with primary antibodies against the following proteins: HTT (1 : 1 000; D7F7, #5656, cell signaling), GFP (1 : 5 000; GFP-1020, Aves Labs), and *α*-tubulin (1 : 10 000; T5168, Sigma). After washing with PBS for 3 times, secondary antibodies conjugated with horseradish peroxidase (1 : 25 000) from Jackson ImmunoResearch (711-035-152, 715-035-150, and 703-035-155) were incubated, respectively, with blots for 2 hrs at room temperature. Protein bands were finally visualized with the Pierce ECL substrate (Thermo Fisher Scientific).

### 2.6. Statistical Analysis

Results were presented as means ± SD values. Statistical significance (*p* < 0.05) was determined using Student's *t*-test.

## 3. Results and Discussion

### 3.1. Capability of Initiating Translation by Noncanonical ATGs

**Figure 1:**
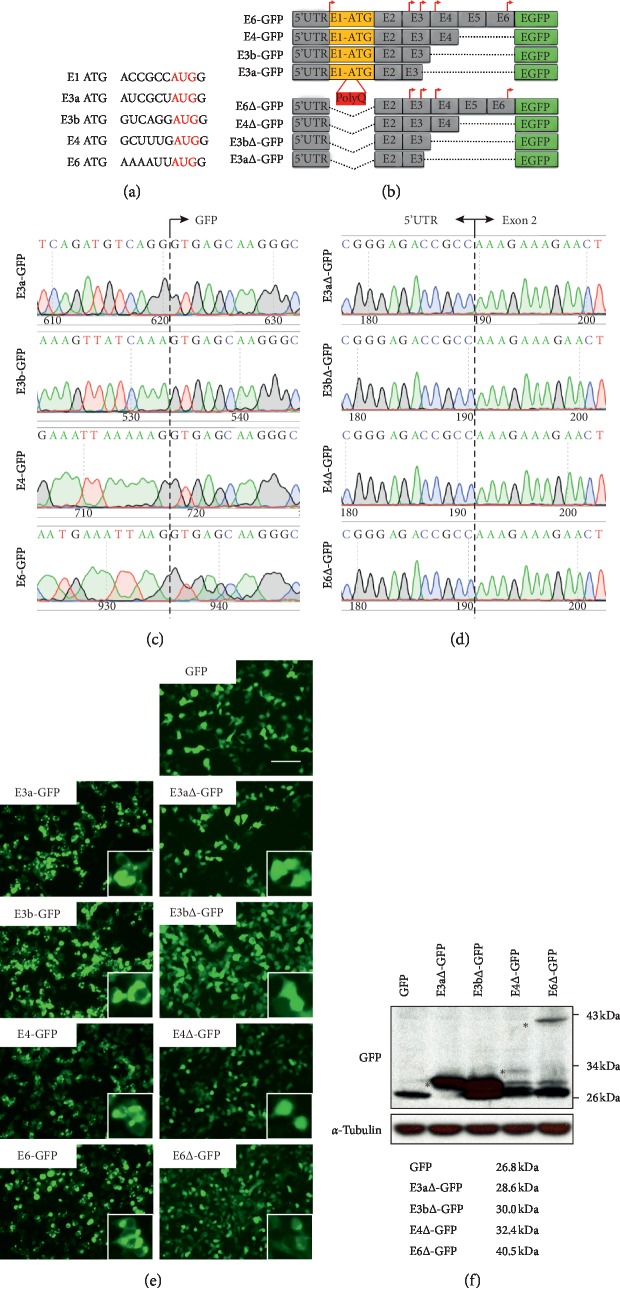
Capability of initiating translation by noncanonical in-frame ATGs. (a) Candidate in-frame ATGs before exon 7 of HTT. (b) Constructs of HTT fragments fused with GFP. (c, d) Sanger sequencing results of junctions in plasmid constructs. (e) Transfection of constructs in HEK293 cells. Scale bar, 100 *μ*m. (f) Western blot analysis of fused proteins. Asterisks represented the E3a-ATG initiated translation products.

Functional truncated HTT is lack of N-terminal 237 amino acids, which are translated from within the first 6 exons [24]. To figure out any possible alternative ATG start codon that may initiate its downstream protein translation and produce functional truncated HTT, we first searched all in-frame ATGs before exon 7. We then identified four candidate ATGs, among which, two are located on exon 3 (E3a-ATG and E3b-ATG), one on exon 4 (E4-ATG), and one on exon 6 (E6-ATG) (Figure 1(a)). Interestingly, these candidate ATGs mostly conform to the consensus Kozak rule NNN(A/G)NNATGG for eukaryotic cells, suggestive of strong initiation potentials (Figure 1(a)).

To test which of these ATGs can indeed start downstream protein translation, we then constructed different lengths of HTT fragments harboring candidate ATGs, respectively, and fused them with GFP in plasmids (Figure 1(b)). Constructs of E3a-, E3b-, E4-, and E6-GFP included exon 1 and thus preferentially used E1-ATG to start their translation. However, exon 1 and E1-ATG were all deleted in E3a∆-, E3b∆-, E4∆-, and E6∆-GFP constructs (Figure 1(b)). All constructs retained the 5′ untranslated region (5′ UTR) of HTT to maintain the upstream regulatory machinery. Sanger sequencing of those constructs confirmed that ATG codons of GFP were all removed (Figure 1(c)) and that 5′UTR and exon 2 were seamlessly ligated in truncated constructs (Figure 1(d)).

**Figure 2:**
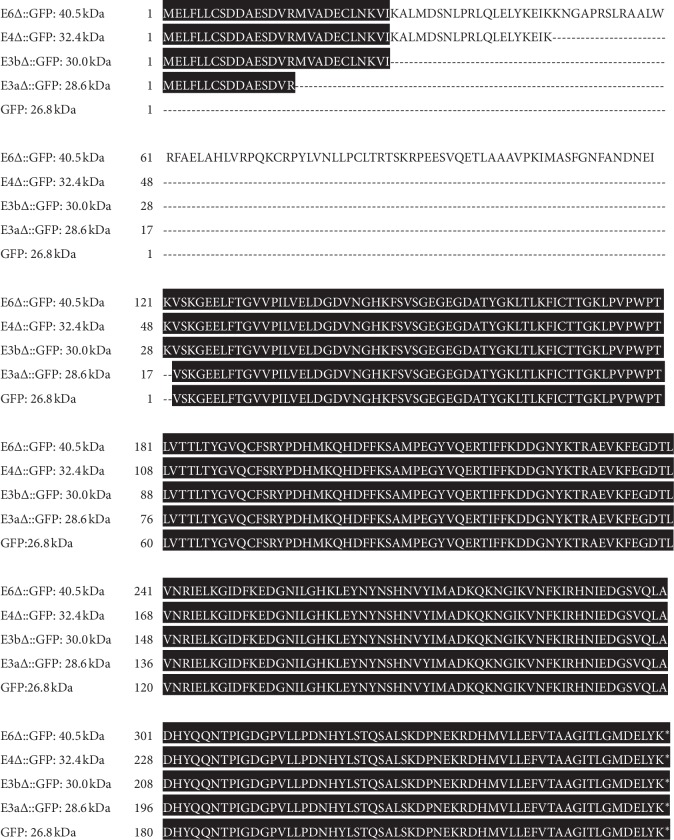
Translation products of HTT : GFP constructs initiated by E3a-ATG. Translation ORFs were aligned using EMBL-EBI multiple sequence alignment program (T-Coffee, http://ww.ebi.ac.uk/Tools/msa/tcoffee/) and viewed by BoxShade Server (https://embnet.vital-it.ch/software/BOX_form.html).

After transfected into HEK293 cells with the same amount, constructs containing exon 1 did produce strong GFP signals and also led to extensive polyQ aggregations in the cytoplasm (Figure 1(e)). This is in line with previous studies that the NES located on the N17 region of exon 1 exported HTT from the nucleus to cytoplasm [14]. Interestingly, all of the truncated constructs (E3a∆-, E3b∆-, E4∆-, and E6∆-GFP) can produce GFP signals evenly in the nucleus and cytoplasm, as those in the GFP-alone control group (Figure 1(e)). Furthermore, western blot analysis showed that different lengths of HTT fragments were successfully translated and fused with downstream GFP although the productivities of E4∆-GFP and E6∆-GFP were greatly decreased (Figure 1(f)). Based on the sizes of protein bands (28.6 kDa for E3a∆-GFP, 30.0 kDa for E3b∆-GFP, 32.4 kDa for E4∆-GFP, and 40.5 kDa for E6∆-GFP), we demonstrated that E3a-ATG is responsible for the translation of those fused proteins (Figure 1(f) and Figure 2). The unspecific bands were probably derived from proteolytic cleavage of translated peptides. Taken together, these results suggest that alternative in-frame ATGs can initiate downstream GFP expression in plasmids.

### 3.2. Targeting N-Terminal HTT with a Dual-sgRNA Strategy

N-Terminal HTT is transcribed from exon 1 just after 5′UTR, containing the conserved N17 region, polyQ, and polyproline (polyP) tract. In light of the initiation capability of downstream ATGs in plasmids, we proposed to endogenously delete the polyQ tract from N-terminal HTT by CRISPR/Cas9, in hope of maintaining the functions of remaining HTT peptides.

**Figure 3:**
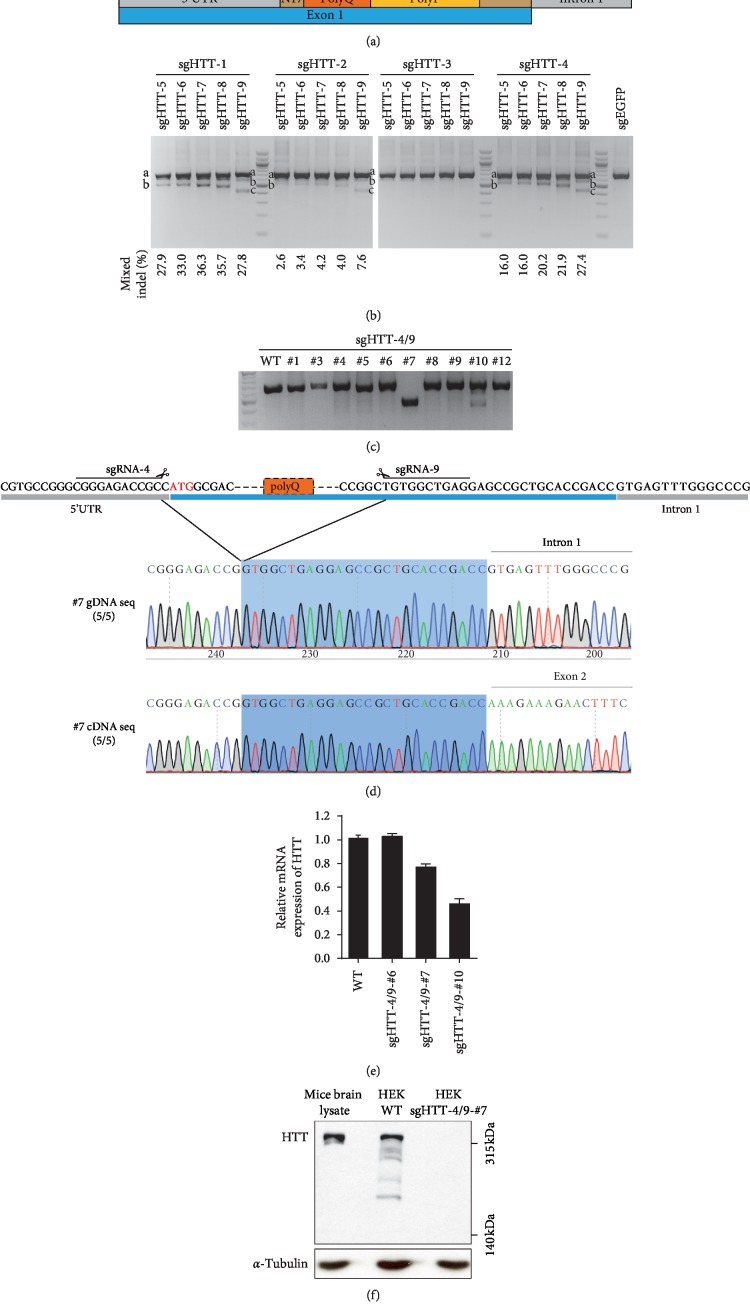
Targeting N-terminal HTT with a dual-sgRNA strategy. (a) Design of sgRNAs flanking the polyQ region. (b) Detection of cleavage efficiencies with different combinations of sgRNA pairs by the T7E1 assay. Mixed indels were calculated based on the fractions of PCR products. “a” represents the undigested PCR product, and “b” and “c” represent cleavage products. (c) Single colonies screening by junction PCR after sgHTT-4/9 targeting. (d) Sanger sequencing results of gDNA and cDNA junctions in the sgHTT-4/9-#7 cell colony. (e) Measurement of HTT levels by quantitative PCR. (f) Western blot detection of full-length HTT and possible truncated HTT proteins.

First, we designed a range of sgRNAs flanking the polyQ region, with 4 upstream sgRNAs and 5 downstream sgRNAs (Figure 3(a) and Table 1). We then used different combinations of sgRNA pairs to transfect cells and later examine their combined cleavage efficiencies by the T7E1 assay. Although this analysis would be a mixture of independent cleavage events induced by each single sgRNA and deletion events induced by the dual sgRNAs, we can nevertheless figure out the sgRNA pairs with higher cleavage efficiencies. Of those, upstream sgHTT-1 and sgHTT-4 combined with downstream sgRNAs were shown to be highly efficient in cutting genomic DNA than those with sgHTT-2 and sgHTT-3 (Figure 3(b)). To retain 5′UTR regulatory elements at the most and avoid the possible regulatory interference by uncut polyP sequences, we thus chose sgHTT-4/9 as the optimized sgRNA pair. We then transfected HEK293 cells and later screened out 12 single colonies. We did junction PCR and finally selected one cell colony (sgHTT-4/9 #7), which is shown to be homozygous, for further analysis (Figure 3(c)). We then sequenced #7 gRNA and found that polyQ was seamlessly deleted from N-terminal HTT (Figure 3(d)).

Since the downstream sgHTT target sites are adjacent to the exon 1/intron 1 boundary, this may disrupt the exon/intron junction by indels and then affect the RNA splicing process. A previous study has demonstrated that targeting the junction of HTT exon 1/intron 1 with CRISPR/Cas9 can reduce half of the mRNA level in the YAC128 mouse model [39]. We thus harvested mRNAs from cell colonies and reverse-transcribed them into cDNAs for integrity analysis. We sequenced #7 cDNA and found that the junction of exon 1/exon 2 was still intact, suggesting that no indels occurred in this region (Figure 3(d)). We next did quantitative PCR, and the results showed a ∼23% reduction of the HTT level in colony #7 and a ∼54% reduction in colony #10 (Figure 3(e)). This result echoes the heterozygous property of colony #10 but, meanwhile, documents that dual-sgHTT targeting in this colony may destroy HTT transcripts by certain mechanisms such as nonsense-mediated decay (NMD) that will eliminate mRNAs containing premature stop codons [42]. Moreover, sgHTT-9 targeting may have produced indels to disrupt the exon 1/intron 1 junction in colony #10, finally dampening the transcription.

We hence extracted proteins and performed western blot to examine the HTT expression. We can observe full-length HTT band and also the cleaved fragments in WT HEK293 cells (Figure 3(f)). Unexpectedly, in the sgHTT-4/9-#7 cell colony, there were no obviously visible HTT bands including any possible truncated HTT bands, suggesting the ineffective or extremely weak initiation capability of E3a-ATG in this endogenous condition (Figure 3(f)). Note that the primary antibody against HTT was produced by immunizing animals with a synthetic peptide corresponding to residues surrounding Pro1220 of human HTT protein, and it is unlikely that truncated HTT proteins cannot be recognized by this primary antibody.

Collectively, our results suggest that downstream noncanonical ATGs may not be effective in the genomic context, which is quite different from their capability in plasmid constructs. Several factors might be accounted for this discrepancy, leading to a failed proof of concept to exploit the possible functions of truncated HTT. First, transient transfection of HTT-exons-GFP constructs basically results in really high-level expression of proteins although we only transfected small amounts of plasmids. Also, CMV is a very strong promoter in constructs that may accelerate this massive expression and lead to the formation of cytoplasmic polyQ aggregates. Notably, the translated WT polyQs are within truncated peptides but not within the full-length HTT protein. In this truncated context, the conformation of translated peptides harboring WT polyQ might be changed and leads to form aggregates more easily. On the contrary, transfection of the E3a∆-GFP construct with this strong promoter only produced small amount of proteins. This partially suggests that E3a-ATG is indeed far less efficient than the canonical E1-ATG start codon. Similarly, in the native genomic context, noncanonical E3a-ATG also has far lower efficiency to produce enough truncated proteins to be discoverable by the western blot assay, and in this case, the potency of truncated HTT would also be far below therapeutic requirements. Second, the complexity of endogenous regulatory system flanking N-terminal HTT is really different from the simple well-known plasmid elements. A more comprehensive analysis of endogenous regulatory elements and their orchestration need to be considered.

By using the knock-in technology with the CRISPR system, further studies may perform gene engineering to keep the strong E1-ATG after 5′UTR that directly ligates with downstream in-frame HTT sequences excluding CAG repeats. Alternatively, it might be possible to drive translation from the E3a-ATG by adding some upstream regulatory elements. And for sure, pathogenic N-terminal HTT can also be replaced by a WT fragment with the similar knock-in method to correct the mutations. The concern is that the efficacy of knock-in mediated by homologous recombination (HR) is much lower than that by nonhomologous end joining (NHEJ). For in vivo gene targeting or gene therapy, it may not be optimal to deliver a stretch of DNA donor together with CRISPR vectors. In this study, we originally aimed to figure out noncanonical ATGs that may initiate truncated HTT translation, in which case we can turn detrimental HTT directly into functional ones only by NHEJ mechanisms. Since E3a-ATG appears not to be strong enough in the genomic context, these knock-in strategies currently may not be promising for in vivo gene therapy. However, these generated knock-in cells would be helpful for future studies in dissecting truncated HTT functions. Overall, our study examined the therapeutic efficacy of downstream noncanonical ATGs for HTT translation and also provided an optimized dual-sgRNA strategy for further genome manipulation of the HTT gene.

## Figures and Tables

**Table 1:** Sequences of sgRNAs and PCR primers.

	Sequences (5′-3′)
*sgRNA sequences*	
sgHTT-1	GAGTCGGCCCGAGGCCTCCG
sgHTT-2	GCCTCCGGGGACTGCCGTGCC
sgHTT-3	GCGGGGACTGCCGTGCCGGGC
sgHTT-4	GTGCCGGGCGGGAGACCGCCA
sgHTT-5	GTGAGGAAGCTGAGGAGGCGG
sgHTT-6	GGCTGAGGAAGCTGAGGAGG
sgHTT-7	GGCGGCGGCTGAGGAAGCTG
sgHTT-8	GAGCAGCGGCTGTGCCTGCGG
sgHTT-9	GAGCGGCTCCTCAGCCACAGC

*T7E1 assay and junction PCR*	
HTT-T7E1-F	CCAGCCATTGGCAGAGTCCG
HTT-T7E1-R	TTGCTGGGTCACTCTGTCTCTG

*Quantitative PCR*	
HTT-qF	TGACGCAGAGTCAGATGTCAG
HTT-qR	CCGAGGGGCACCATTCTTTT
GAPDH-qF	CGGGATTGTCTGCCCTAATTAT
GAPDH-qR	GCACGGAAGGTCACGATGT

*Sequencing of junctions*	
HTT-5′UTR/e1-F	CGGGAGACCGGTGGCTGAGG
HTT-e4/e5-R	CCGAGGGGCACCATTCTTTT

## Data Availability

The data used to support the findings of this study are available from the corresponding author upon request.
